# Preparation of Multifunctional Surfactants Derived from Sodium Dodecylbenzene Sulfonate and Their Use in Oil-Field Chemistry

**DOI:** 10.3390/molecules28083640

**Published:** 2023-04-21

**Authors:** Yongfei Li, Quanzheng Bai, Qiang Li, Hai Huang, Weijun Ni, Qian Wang, Xin Xin, Bin Zhao, Gang Chen

**Affiliations:** 1 Shaanxi University Engineering Research Center of Oil and Gas Field Chemistry, Xi’an Shiyou University, Xi’an 710065, China; 2 Xi’an Changqing Chemical Group Co., Ltd., Xi’an 710018, China; 3 Shaanxi Province Key Laboratory of Environmental Pollution Control and Reservoir Protection Technology of Oilfields, Xi’an Shiyou University, Xi’an 710065, China; 4 Department of Crop Soil Sciences, Washington State University, Pullman, WA 99163, USA; 5 Department of Statistics, North Dakota State University, Fargo, ND 58102, USA

**Keywords:** sodium dodecylbenzene sulfonate, multifunctional surfactant, interfacial tension, foaming, emulsification, oil displacement

## Abstract

Four products were obtained from sodium dodecylbenzene sulfonate (SDBS) and formaldehyde (40% solution) using a simple reaction. The products were characterized by TGA, IR, UV and MS to confirm the major chemicals in each sample. The new products could reduce the interfacial tension between oil and water in the experimental temperature range further compared to SDBS. The emulsion ability was also enhanced by SDBS-1 to SDBS-4. The oil-displacement efficiencies of SDBS-1 to SDBS-4 were obviously higher than that of SDBS, and the oil-displacement efficiency of SDBS-2 was the best, with an efficiency of 25%. The experimental results all indicate that these products have an excellent ability to reduce oil–water interfacial tension and that they can be used in the oil and petrochemical industry for oil production and have certain practical uses.

## 1. Introduction

Surfactants have been widely used in daily life, industrial production and high-tech fields as some of the most important industrial additives, known as “industrial monosodium glutamate”. Surfactants are the finishing touch in many industries, where a small amount can significantly improve the physical and chemical properties of the surface (interface) of a substance [1,2,3].

The important components in the liquid system of oil field chemistry are directly or indirectly related to surfactants, and their properties and functions depend on the development and application levels of surfactant technology to a large extent. Therefore, surfactants play an important role in oil field chemical application technology and working liquid systems and can even be said to be one of the key factors for the success of oilfield chemical and application technology research and development. During oil recovery, displacement efficiency, interfacial tension between crude oil and the system and emulsification and demulsification of crude oil are all related to surfactants. When a surfactant is dissolved in water, the interfacial tension is reduced due to its interfacial adsorption, and the permeability, emulsification and dispersion are also significantly changed compared with those of pure water [4,5,6]. Appropriate surfactants can even reduce the interfacial tension between oil and water to 10^−3^–10^−5^ m N/m, resulting in significantly improved oil recovery. Therefore, the field of surfactant research has been relatively active [7,8,9].

The application and production of surfactants provide a new idea for the study of oil reserves and enhanced oil recovery and lay a solid foundation for long-term development of oil fields. However, in the complex formation environment, due to its special structure, the surfactant performance is lower than the theoretical value, so the efficiency is reduced. Therefore, in order to be efficient and economical, multifunctional surfactants will need to be developed to be used for foaming and emulsifying, as well as for reducing surface interfacial tension to achieve oil displacement. At present, anionic sulfonate and nonionic surfactants are the most commonly used surfactants in field experiments [10,11,12,13]. However, nonionic surfactants cannot reach the appropriate standard in the complex and diverse high-salinity reservoir environment. Anionic sulfonate surfactants can make crude oil pass through pores smoothly under the condition of high salinity so as to improve its solubility and recovery efficiency, and as a thermodynamically stable system, it can also be placed for a long time without a settling phenomenon. Therefore, the research idea of this paper is to start with this type of surfactant, sodium dodecyl benzene sulfonate and different amounts of formaldehyde (40% solution) through an Aldol reaction to prepare new surfactants. The mechanism is to introduce one or two hydroxyl methyl groups in the lateral position of the benzene-ring sulfonate group so that it not only has a nonionic hydrophilic group but also retains the advantages of anionic sulfonate. The introduction of methylol enhances the foaming and emulsifying capacities of sodium dodecyl benzene sulfonate and further reduces the interfacial tension between crude oil and water. This study achieved the goal of multi-effect and multi-use of one dose and further expanded the practical application range.

## 2. Results and Discussion

It should be noted that four products were obtained (denoted as SDBS-1 to SDBS-4), probably mixtures of SDBS with its products of hydroxylation, with the degrees of hydroxylation increasing from SDBS-1 to SDBS-4.

### 2.1. Thermogravimetric Analysis

**Figure 1:**
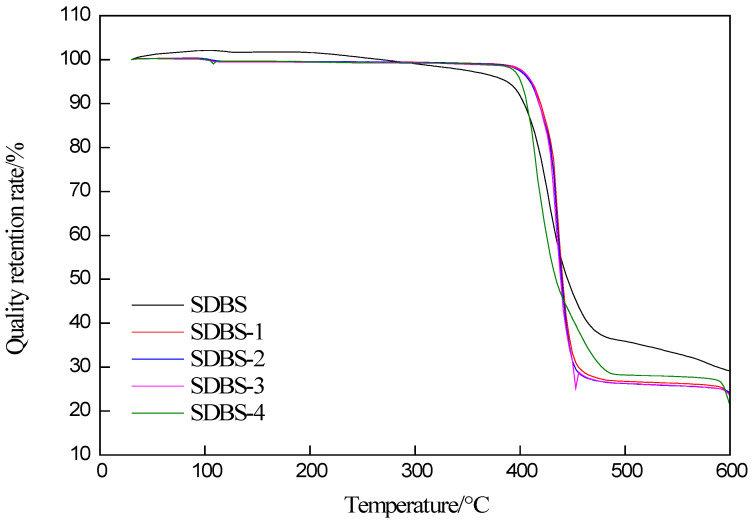
TG of SDBS to SDBS-4.

Thermogravimetric analysis of SDBS, SDBS-1, SDBS-2, SDBS-3 and SDBS-4 was conducted, as shown in Figure 1. It can be seen from the temperature graph (TG) in Figure 1 that the SDBS begins to lose weight slowly at 200 °C, intensely at 400 °C and slowly at 480 °C. SDBS-1 to SDBS-4 begin to lose weight at 410 °C and basically end at 470 °C. The weight loss process was much faster than that of the SDBS, and there was only one stage in this weight loss process. After the SDBS’s weightlessness, about 30% of the residue was left, while SDBS-1 to SDBS-4 had about 23% residue. The reason why the temperatures of SDBS-1 to SDBS-4 at the beginning of weightlessness were slightly lower than that of the SDBS is that the introduction of hydroxymethyl added another carbon to the molecular branched chain, and the hydroxyl may have also formed intermolecular hydrogen bonds, thereby improving the thermal stability of the molecules [13,14,15].

### 2.2. Infrared Analysis

**Figure 2:**
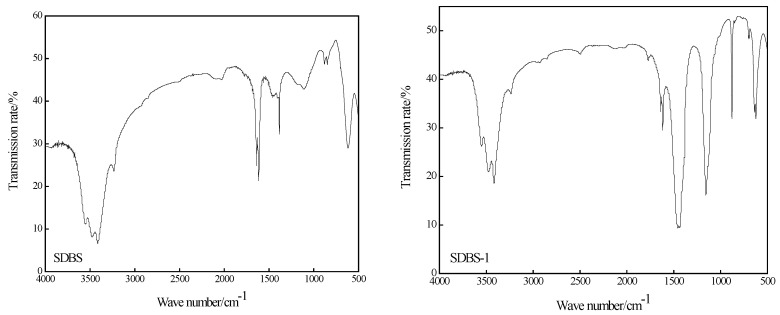

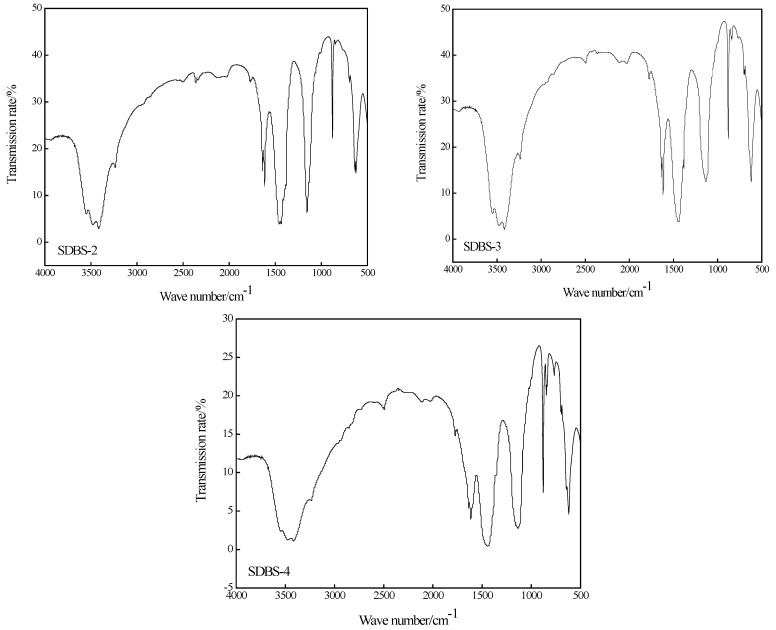
FT-IR of SDBS to SDBS-4.

Infrared characterizations of SDBS, SDBS-1, SDBS-2, SDBS-3 and SDBS-4 were carried out. The spectra are shown in Figure 2. As shown in Figure 2, SDBS-1 to SDBS-4 have broad and strong absorption peaks from about 3550 to 3100 cm^−1^. At this time, the characteristic absorption peaks of the free and associative hydroxyl groups overlap, indicating that these products all contain hydroxyl groups. At the same time, it can be seen that the spectra of SDBS-1 to SDBS-4 are obviously different from those of the SDBS. The absorption peaks of SDBS are very narrow, at about 1800 and 800 cm^−1^, and the absorption peak at 800 cm^−1^ is weak, which is a typical characteristic absorption peak of paradisubstituted benzene [15]. The infrared spectra of SDBS-1 to SDBS-4 show absorption peaks at about 1870 cm^−1^ and 1650 cm^−1^ and corresponding absorption peaks at about 800 and 850 cm^−1^, especially for SDBS-4, which has a typical characteristic absorption peak for triple-substituted benzene. At the same time, the wave number of each absorption peak is smaller than the theoretical value. This is because the hydroxymethyl group was an electron-donor group that caused a red shift in the absorption wavelength and decreased the wave number [16]. All of these indicate that the expected products were obtained.

### 2.3. Ultraviolet Spectroscopy

**Figure 3:**
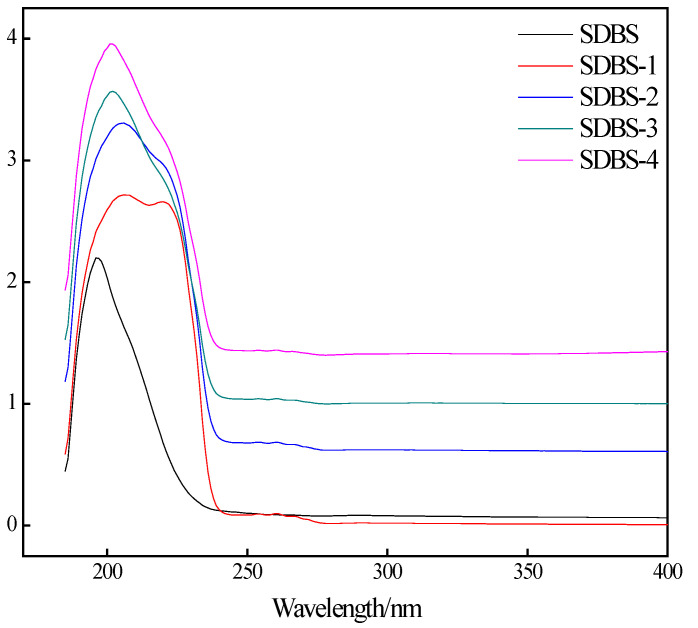
UV of SDBS to SDBS-4.

Ultraviolet characterizations of SDBS, SDBS-1, SDBS-2, SDBS-3 and SDBS-4 were performed, and the spectra are shown in Figure 3. It can be seen from Figure 3 that the SDBS series has similar ultraviolet absorption peaks, except SDBS-1, and all have strong absorption peaks at 220 nm, which is a typical absorption peak for the π-π* transition of the C=C bond. In addition, the other four surfactant absorption values are higher than that of the SDBS, and the wavelength at the peak is slightly smaller than that of the SDBS, possibly due to the introduction of hydroxymethyl. Hydroxymethyl is a typical electron-donor group that is superconjugated when attached to a benzene ring, resulting in a blue shift at the maximum absorption wavelength [17]. In addition, SDBS-1 also has an absorption peak at about 230 nm. This may be caused by the n-σ* transition of the S=O bond.

### 2.4. Mass Spectrum

**Figure 4:**
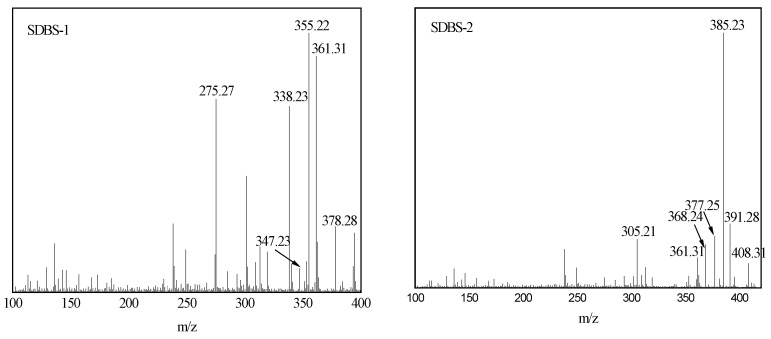
MS of SDBS-1 and SDBS-2.

The mass spectrum was the same, since the molecular ion peaks of SDBS-2, SDBS-3 and SDBS-4 were substantially equal. Therefore, only the spectra of SDBS-1 and SDBS-2 are listed in Figure 4. Figure 4 shows that *m*/*z* 378.28 is the molecular ion peak of SDBS-1, *m*/*z* 361.31 is the peak of [M-OH], *m*/*z* 355.22 is the peak of [M-Na], *m*/*z* 347.23 is the peak of [M-CH_2_OH], *m*/*z* 338.23 is the peak of [M-OH-Na] and *m*/*z* 275.27 is the peak of [M-SO_3_Na], respectively. As shown in Figure 4, *m*/*z* 408.31 is the peak of SDBS-2, *m/z* 391.28 is the peak of [M-OH], *m*/*z* 385.23 is the peak of [M-Na], *m*/*z* 377.25 is the peak of [M-CH_2_OH], *m*/*z* 368.24 is the peak of [M-OH-Na] and *m*/*z* 305.21 is the peak of [M-SO_3_Na], respectively. All of these indicate that the reaction produced the target product.

### 2.5. Surface-Tension Evaluation

**Figure 5:**
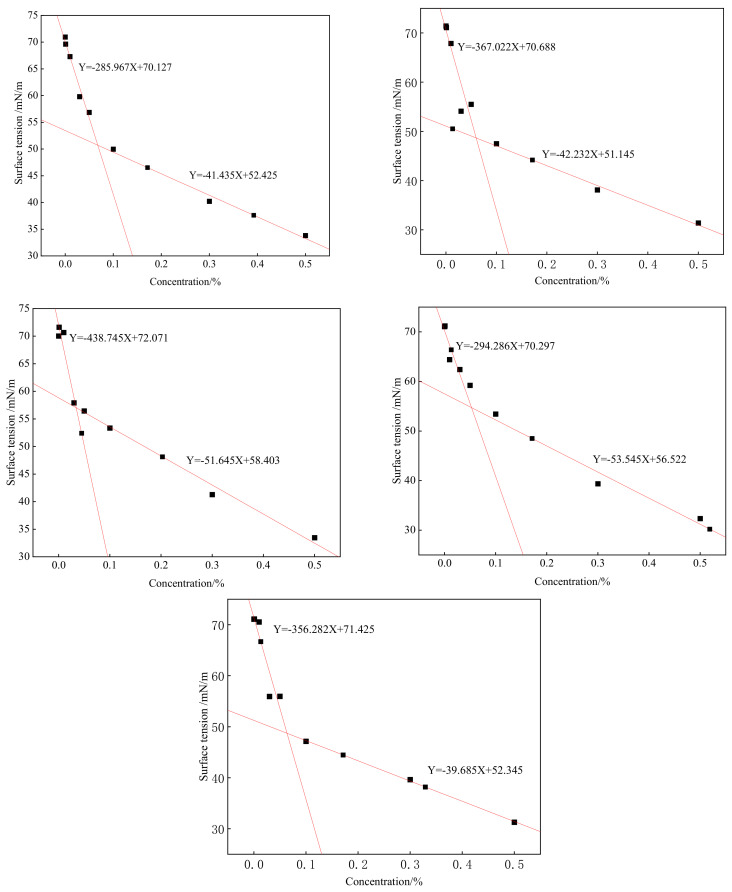
Surface tensions of SDBS to SDBS-4.

Surfactant solutions with mass fractions of 0.0001, 0.001, 0.01, 0.03, 0.05, 0.1, 0.3 and 0.5% were prepared using distilled water, the surface tension of each solution was measured with a loop method and the results are shown in Figure 5. It can be seen from Figure 5 that with increasing concentration, the surface tensions of the SDBS to SDBS-4 solutions first drop rapidly, and then the speed slowly descends, with an inflection point in the middle. This is because molecules can be aligned in an aqueous solution. Within a certain range, the higher the concentration was, the more molecules would be deposited on the surface of the solution, and the smaller the corresponding surface tension would become; however, as soon as the liquid surface was completely covered, increasing the concentration in the solution caused corresponding counterions to aggregate and form micelles [18,19,20]. At that point, the surface tension of the solution tended to be stable. 

The data in Figure 5 was processed to obtain the critical micelle concentrations (CMC) of the SDBS to SDBS-4 surfactants [21], which are shown in Table 1. From this result, it can be seen that the differences between the critical micelle concentrations of the SDBS-series surfactants are very large, especially for SDBS-2, in which the critical micelle concentration is much smaller than those of the other four and the methylol group is introduced due to the difference in the amount of formaldehyde (40% solution). The amount affected the critical micelle concentration.

### 2.6. Interfacial-Tension Evaluation

**Figure 6:**
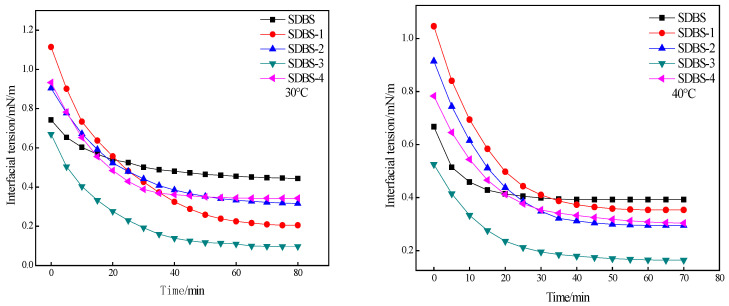

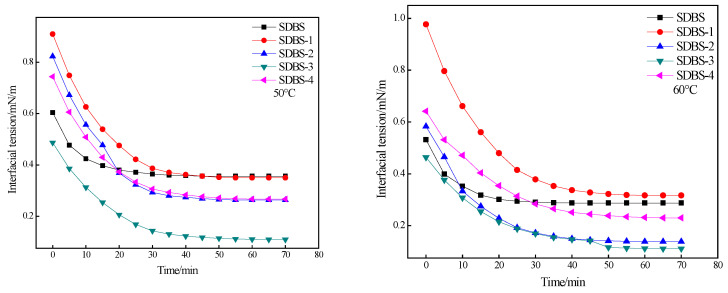
Interfacial tension of SDBS to SDBS-4.

The interfacial tensions were evaluated for SDBS, SDBS-1, SDBS-2, SDBS-3 and SDBS-4. The experimental results are shown in Figure 6. It can be seen from Figure 6 that at 30 °C, the SDBS interfacial tension decreases little with time, only from 0.8 mN/m to about 0.5 mN/m. Although SDBS-1, SDBS-2 and SDBS-4 initially have interfacial tensions greater than that of the SDBS, the final values are lower than that of the SDBS, and the SDBS-3 interfacial tension is always lower than that of the SDBS, indicating that temperature, in relation to SDBS and SDBS-1 to SDBS-4, can effectively reduce oil–water interfacial tension. In order to better compare the abilities of the three and reduce oil–water interfacial tension, the temperature was increased to 40, 50 and 60 °C, respectively, and the oil–water interfacial tension was measured at the abovementioned temperature. In the experimental temperature range, the SDBS interfacial tension began to be scheduled. When the time was reduced to ~0.7 mN/m, the final reduction was not large. At the beginning of the measurement, the initial values of interfacial tension of SDBS-1 to SDBS-4 were between 0.8 and 1.0 mN/m, but they were all lower than that of the SDBS, indicating that their temperature resistance is better than that of SDBS. At the same time, at 40 and 50 °C, the interfacial tensions of SDBS-1 and SDBS-2 decreased with time. During the temperature changes, the interfacial tension of SDBS-4 decreased relatively slowly, but the interfacial tension was always less than those of the SDBS to SDBS-3. Since the ionicity of SDBS itself weakens when a methylol group is introduced into the SDBS molecule, the influence of calcium and magnesium ions on the interfacial tension of water is also small. Therefore, the influence of temperature on the abilities of the SDBS to SDBS-4 to reduce the oil–water interface is complex. Figure 6 shows a tendency to decrease first and then increase. This may be the result of the joint effect of temperature rise on solubility and adsorption power at the oil–water interface [22]. Combined with the results of the surface-tension test, it was found that the lower the surface tensions of the surfactants were, the stronger the ability to reduce oil–water interfacial tension was. Therefore, it can be considered that SDBS-1 and SDBS-2 in the SDBS-series surfactants have stronger abilities to reduce oil–water interfacial tension.

### 2.7. Foaming-Ability Test

**Figure 7:**
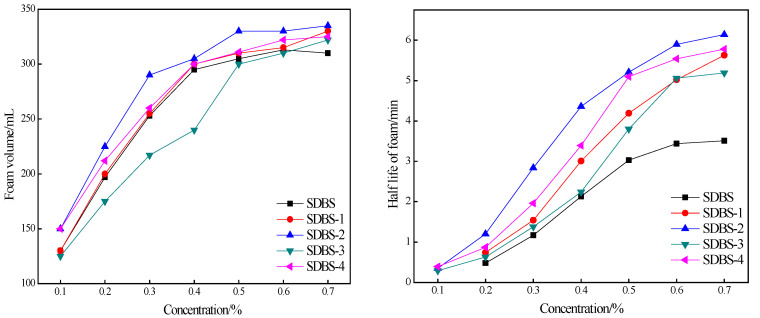
Foaming-ability tests (stirring method) for SDBS to SDBS-4.

Foam also plays an important role in enhancing oil recovery. A stable foam system can have an oil-displacement effect similar to that of gel and has a lower cost. SDBS is also one of the main surfactants used in foam flooding. The foaming abilities of SDBS, SDBS-1, SDBS-2, SDBS-3 and SDBS-4 were tested, and the experimental results are shown in Figure 7. First, it can be seen from Figure 7 that with an increase in concentration, the volumes and half-lives of the SDBS to SDBS-4 foams also increase, but that increase becomes smaller and smaller. There is little difference between the SDBS-1 to SDBS-4 foam height and that of the SDBS. The SDBS-3 foam volume is less than that of the SDBS, but the SDBS-1 to SDBS-4 foam half-lives are much higher than that of the SDBS, indicating that their foaming capacities are better than that of SDBS. Secondly, as shown in Figure 7, the foam-volume and half-life curves of SDBS-2 are always the highest in the SDBS series. Considering the foam volume and half-life, the optimum foaming concentration of the SDBS-series surfactants should be 0.7%. At this concentration, the foam half-lives of SDBS-1 to SDBS-4 are higher than that of SDBS, and SDBS-2 has the best foaming ability.

**Figure 8:**
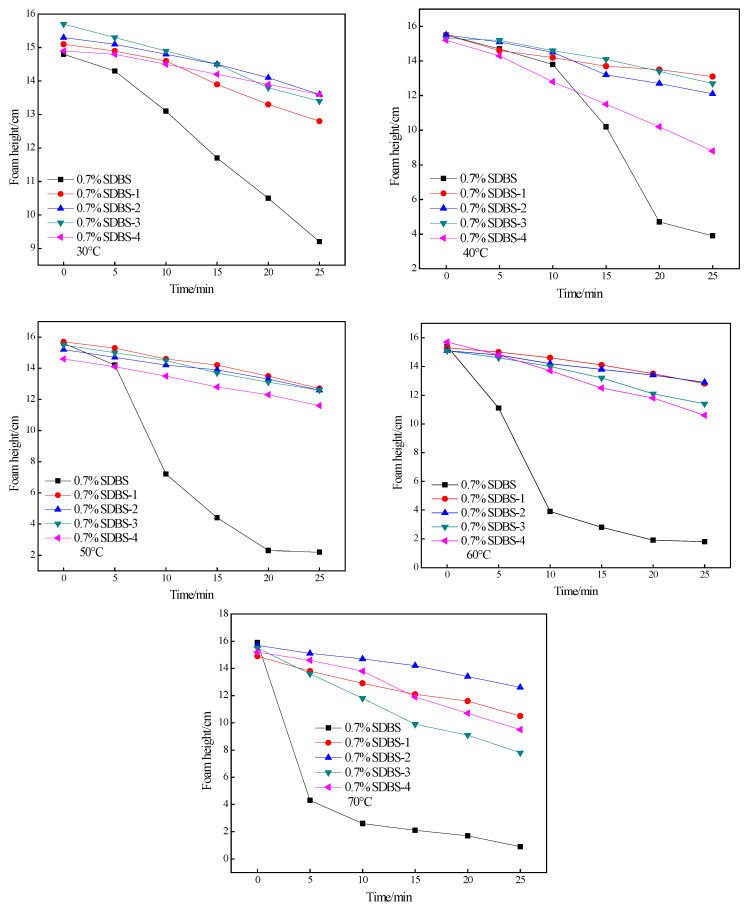
Foaming ability tests (Ross–Miles foam) for SDBS to SDBS-4.

Furthermore, the foaming abilities of the SDBS to SDBS-4 at different temperatures were measured with a Roche foam meter to evaluate foam stability. The experimental results are shown in Figure 8.

As shown in Figure 8, as the temperature increases, the initial foam height of the SDBS to SDBS-4 increases because the activity of the surfactant increased with increasing temperature over a certain temperature range. At the same time, the rate at which the foam is highly attenuated increases, because as the temperature rose, the rate of liquid loss in the foam increased, and the rate at which the gas diffused through the liquid film also increased. As a result, these two effects led to an increase in the breaking speed of the foam [23]. At the same time, the stabilities of different surfactant foams were also different. It can be seen from Figure 8 that SDBS-1 to SDBS-4 are much larger than the SDBS in the experimental temperature range at low temperature, whether in foam height or foam stability. This advantage is still evident when the experimental temperature increases gradually. The reason for the above phenomenon is due to the effect of temperature on foam decay: at low temperatures, the gas-diffusion rate in the foam was slower; the liquid in the foam continued to drain, causing its wall to become thinner; and the stability became worse. The mechanism of this decay was mainly the diffusion of gas in the foam. Together, they formed a larger bubble that was reduced in volume faster than the drainage [24]. At the same time, the bubble wall was a bending film that was very sensitive to evaporation. With an increase in temperature, the movement of the gas molecules increased, hence the faster diffusion rate. On one hand, the bubble film became thinner. On the other hand, the bubbles became larger and larger. These two functions caused the bubbles to collapse [25]. As shown in Figure 8, the decay of the SDBS to SDBS-4 foam was dominated by liquid drainage.

### 2.8. Microstructure Analysis of Foam

**Figure 9:**
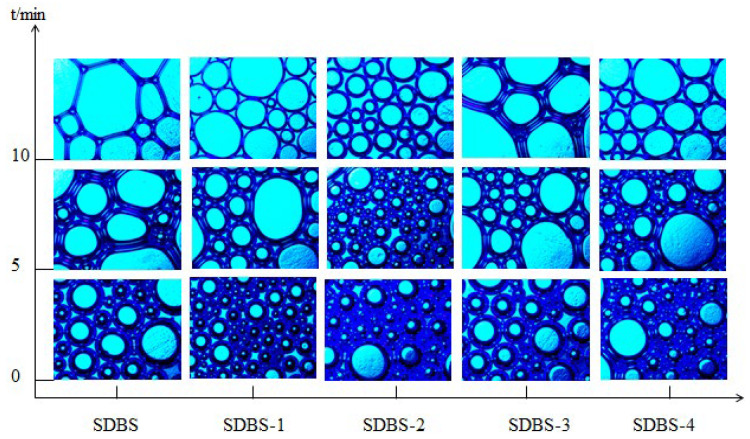
Variation of foam microstructures with time for SDBS to SDBS-4.

Optical microscopy was used to observe changes in the foam microstructures of SDBS to SDBS-4 with time. Polarized light was selected as the light source. The experimental results are shown in Figure 9.

In order to better explore the foaming ability of each surfactant, the foam characteristic value Φ was introduced, referring to the ratio of the foam’s gas volume to the total volume of the foam. When the Φ was in the range of 0.52–0.74, the water content of the foam was larger than that of the wet foam, and the foam was spherical or ellipsoidal. As the liquid in the foam continued to precipitate, the Φ increased continuously, and when it was greater than 0.74, it became dry foam [26]. The foam was generally polygonal, and the Φ values of the SDBS to SDBS-4 foams at different times are listed in Table 2.

It can be seen from Figure 9 and in combination with Table 2 that the SDBS to SDBS-4 foams have a spherical shape at 0 min, when the foam has just been formed and contains more water. At 10 min, only the SDBS foam shape becomes a distinct polygon. The foams of the other four surfactants are still spherical, and the SDBS-1 foam wall is the thickest, that of SDBS-4 is the second-thickest and that of SDBS-2 is the thinnest. Generally speaking, the thicker the foam wall is, the better the mechanical strength and stability are. In addition, the stability of the foams was also related to viscosity, and viscosity is also related to eigenvalue [16], which can be expressed by the following:

(1) $$\eta =\eta_{0}(1.0+4.5\Phi )\;\;(0.52<\Phi <0.74)$$

(2) $$\eta =\eta_{0}\left[\frac{1}{1-\Phi^{1/3}}\right]\;\;(\Phi >0.74)$$

In general terms, the greater the viscosity of the foam is, the stronger the stability is. It can be seen from the above formulas that the viscosity of wet foam is linearly related to the characteristic value of the foam, while the relationship between dry foam and Φ is exponentially potent. The viscosity of both increases with an increase in Φ, and the increase for dry foam is much greater than that for wet foam [27]. As shown in Table 2, at 0 min, the Φ values of SDBS-1 to SDBS-4 are all greater than that of the SDBS. At 10 min, SDBS-2 > SDBS-4 > SDBS-1 > SDBS-3 > SDBS and the foam viscosity order is also SDBS-2 > SDBS-4 > SDBS-1 > SDBS-3 > SDBS, hence the foam stability order is SDBS-2 > SDBS-4 > SDBS-1 > SDBS-3 > SDBS. Ideally, when foam is formed, the energy (surface energy) of the system increases correspondingly with increases in liquid surface area. When the foam breaks, the energy of the system also decreases correspondingly. At the same time, it can be seen from the energy point of view that low surface tension is beneficial to formation of foam. The results of the surface-interfacial-tension test and the 30 °C foaming-ability test show that the smaller the interfacial tension of the surfactant is, the stronger the foaming ability is; that is, lower surface interfacial tension is favorable for foaming.

### 2.9. Emulsification Test

**Figure 10:**
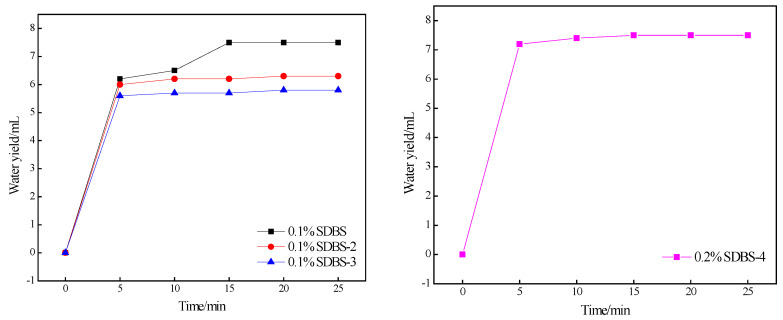
Emulsification ability for SDBS to SDBS-4. (At 0.1%, the water content of SDBS-1 and SDBS-4 is 0 mL, and the water content of other surfactants at 0.2%, except SDBS-4, is 0 mL each).

Crude oil is viscous, mainly composed of mixtures of various alkanes, cycloalkanes and aromatic hydrocarbons. The resin and asphaltene in oil aggregate to form colloidal particles, and crude oil has certain colloidal and gel properties. Surfactant aqueous solutions emulsify crude oil to form O/W lotions, which can significantly improve fluidity of crude oil and thus enhance oil recovery. Emulsification ability tests were carried out on SDBS, SDBS-1, SDBS-2, SDBS-3 and SDBS-4. The experimental results are shown in Figure 10.

At 25 min, the water content of the precipitated water accounted for the total water-volume fraction, and the water-splitting rate is shown in Table 3.

**Figure 11:**
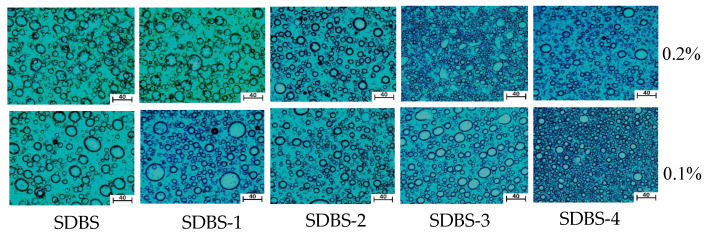
Emulsion microstructures of SDBS to SDBS-4 (magnification was 40 times).

As shown in Figure 11, when the surfactant concentration is 0.1%, the precipitation rates of SDBS-2 and SDBS-3 are obviously slower than that of the SDBS, and the final precipitation water is also less than that of the SDBS; SDBS-1 and SDBS-4 did not precipitate water during the experimental time, illustrating that the SDBS-1 to SDBS-4 emulsification abilities are better than that of SDBS. When the surfactant concentration was 0.2%, with the exception of SDBS-4, the emulsions formed by the other four surfactants did not precipitate water during this experiment, indicating that the emulsifying ability of SDBS-4 is not as good as SDBS at this concentration, and the water was separated, as shown in Table 4. Rate data also led to similar conclusions. Meanwhile, from the water-dissolution-rate data in Table 3, it can be seen that as the concentration of the same surfactant increases, in addition to SDBS-4, the emulsion-precipitation rates of the other four surfactants decrease, which causes this phenomenon. The reason may be the difference in density between crude oil and water. Within a certain range, an increase in the mass fraction of a surfactant increases the diameter of the generated emulsion particles, thereby accelerating the separation of crude oil and water [25]. In addition, when crude-oil droplets float on the surface of a liquid, part of them will aggregate into a mass. At this time, increasing the concentration of the surfactant will increase the self-aggregation between the droplets of the already aggregated cluster, and the result is counterproductive [26].

### 2.10. Microstructure of Emulsion

The microstructures of the SDBS to SDBS-4 emulsions were observed with an optical microscope. Polarized light was selected as the light source. The results are shown in Figure 11. It can be seen From Figure 11 that the particle sizes and distributions of the emulsions formed by the SDBS-series surfactants and the crude-oil samples after oscillation are quite different, but they are spherical, indicating that the SDBS-series surfactants have a great influence on the structure of emulsion droplets. At the same time, it can be seen that only SDBS-4 at a high concentration is greater than that at a low concentration, while the SDBS to SDBS-3 are contrary to SDBS-4. In general, the larger the droplet size of an emulsion is, the faster its coalescence speed will be and the worse its stability, which is consistent with the results of the emulsifying ability test [27].

### 2.11. Oil-Displacement Ability

**Figure 12:**
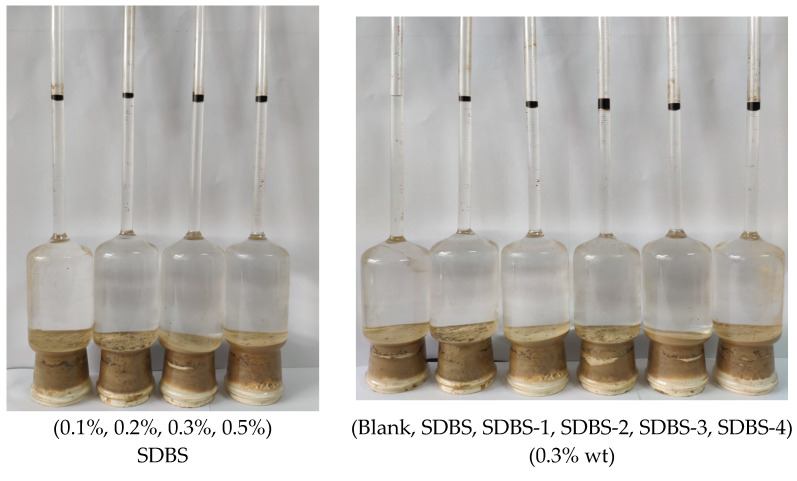
Oil-displacement effect of the SDBS with different concentrations (**left**) and of different surfactants (0.3% wt) (**right**).

The displacement capacities of SDBS, SDBS-1, SDBS-2, SDBS-3 and SDBS-4 were tested. The experimental results are shown in Figure 12. As shown in Figure 12 (left), the oil-displacement effects of SDBS with different concentrations indicate that concentration is an important factor in oil-displacement efficacy. As the concentration rose to 0.3%, the efficiency was much higher than that at 0.1%, and at 0.5%, it was also higher than that at 0.3%. Considering the cost performance, 0.3% was chosen for further research. Figure 12 (right) shows the oil-displacement effect of the SDBS as well as its derived surfactants, and the efficiency is summarized in Table 5, which shows that SDBS-2 has the best displacement efficiency, of 25%, indicating that synthesis of SDBS-series surfactants can be greatly improved. The results show that SDBS-series surfactants (SDBS-1 to SDBS-4) can significantly reduce oil–water interfacial tension and surface tension and effectively improve oil recovery.

## 3. Materials and Methods

### 3.1. Materials

Sodium dodecylbenzenesulfonate (SDBS) was purchased from the Tianjin Hongyan Reagent Factory (Tianjin, China), methanol and potassium bromide were purchased from the Tianjin Tianli Chemical Reagent Group (Tianjin, China), 40% formaldehyde solution was purchased from the Chengdu Kelon Chemical Reagent Factory (Chengdu, China) and crude-oil samples were obtained from an oil field.

### 3.2. Preparation of Multifunctional Surfactant

**Figure 13:**
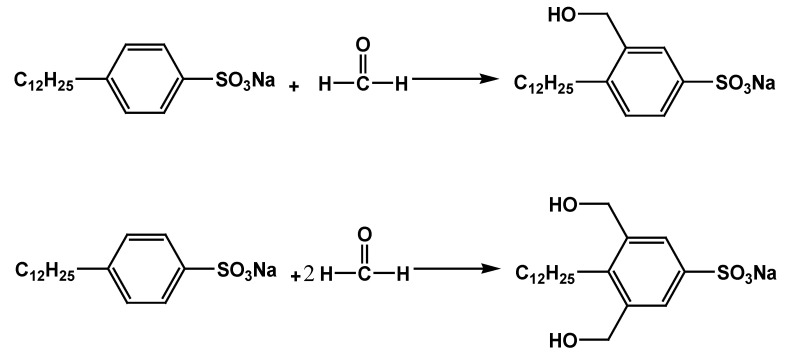
Reaction of sodium dodecylbenzene sulfonate and formaldehyde.

Formaldehyde (40% solution) and sodium dodecylbenzene sulfonate were weighed in proportion (molar ratio). Sodium dodecylbenzene sulfonate was placed in a 250 mL flask, and 50 mL of methanol was added as a solvent. Formaldehyde (40% solution) was added dropwise to the flask at reflux temperature, and acceleration of dropping was controlled for 1 d/s. The reflux temperature was measured for 3 h. After the reaction, the product was obtained with vacuum distillation and the solvent was also removed with vacuum distillation; the reaction formula is shown in Figure 13. As shown in Table 1, different products can be obtained by controlling the ratio of sodium dodecylbenzenesulfonate to formaldehyde (40% solution). 

### 3.3. Thermogravimetric-Analysis Conditions

Thermogravimetric analysis was carried out according to the National Standards of the People’s Republic of China GB/T27761-2011. Surfactants of about 4–10 mg were placed in a crucible of a sample of peeled silicon dioxide and weighed on a sample table of a TGA-DSC thermal analyzer. With nitrogen protection used, the flow rate was 20 mL/min, the heating rate was 10 °C/min, the sampling interval was 0.1 °C and the quality change of each surfactant was recorded at 30–600 °C. 

### 3.4. Infrared-Analysis Conditions

The infrared analysis of the surfactants was in accordance with National Standard GB/T 6040-2002 of the People’s Republic of China. The synthesized product was characterized by infrared spectroscopy using a solid-state method, and the carrier was potassium bromide. 

### 3.5. Ultraviolet-Analysis Conditions

The ultraviolet analysis of the surfactants was in accordance with Mechanical Industry Standard JB/T 6777-1993 of the People’s Republic of China. The synthesized product was characterized using a liquid method (0.1%), and the solvent was distilled water. 

### 3.6. Mass-Spectrometric-Analysis Conditions

The surfactants were analyzed with mass spectrometry according to National Standard GB/T 6041-2002 of the People’s Republic of China. In the mass spectrometric analysis, a nanoelectrospray ionization source, a positive ion mode, a capillary tip size of 1 micron, a collision gas of 99.995% argon, a voltage of 1.2 kV and an ion transfer tube temperature of 270 °C were used. 

### 3.7. Surface-Tension Test Conditions

According to National Standard GB/T 22237-2008 of the People’s Republic of China, the method of determining surface tensions of surfactants was analyzed. Surfactant solutions of 0.0001, 0.001, 0.01, 0.03, 0.05, 0.1, 0.3 and 0.5% (mass fraction) deionized water were used to determine the surface tension of each surfactant solution using the hanging-ring method. 

### 3.8. Test Conditions for Interfacial Tension

The method for determining the interfacial tensions of surfactants was in accordance with National Standard of the People’s Republic of China GB/T 6541-1986 (1991). A 1% (mass fraction) surfactant solution was prepared with deionized water, and kerosene was used as an internal phase, with a rotary drop method. 

### 3.9. Test Conditions for Foaming Capacity

The foaming abilities of surfactants were tested in accordance with National Standard GB/T 7462-1994 of the People’s Republic of China. First, the optimum foaming concentrations of the surfactants were tested with agitation. The experimental temperature was at room temperature, the volume of the solution was 100 mL, the stirring rate was 7000 rad/min, the stirring time was 3 min, the formed foam was immediately poured into a 500 mL measuring cylinder when the stirring was stopped, and a stopwatch was scheduled to record the foam volume and the foam half-life (the precipitation time of 50 mL of water). The foaming abilities of the surfactants at different temperatures were determined with a Roche foam apparatus. 

### 3.10. Microstructure Analysis of Foam

The microstructures and macrostructures of the surfactants were observed with an optical microscope. Polarized light was selected as the light source [28]. 

### 3.11. Test Conditions for Emulsifying Ability

The emulsifying properties of the surfactants were tested according to National Standard GB/T 6369-2008. Surfactant solutions of 0.1% and 0.2% (mass fraction) were prepared, and 10 mL of the above solutions and 10 mL of crude oil were measured, placed in a 25 mL plugged test tube and subsequently placed in a water bath, using an experimental temperature set at 45 °C and at a constant temperature for 10 min. After removal, it was shaken 100 times to form an oil-in-water emulsion, which was then placed in a water bath, and the volume of the precipitated water was recorded every 5 min until the change was small, in addition to the dissolution rate of water that was recorded.

$$\mathrm{Water}\;\mathrm{splitting}\;\mathrm{rate}=\frac{\mathrm{Amount}\;\mathrm{of}\;\mathrm{precipitated}\;\mathrm{water}}{\mathrm{Amount}\;\mathrm{of}\;\mathrm{added}\;\mathrm{water}}\times 100$$

### 3.12. Microstructure Analysis of Emulsion

The microstructures of the surfactant emulsions were observed with optical microscopy, and polarized light was chosen as the light source [29]. 

### 3.13. Test Conditions for Oil-Displacement Capacity

Dried core sand and the experimental oil were uniformly mixed in a ratio of 7:1 (volume ratio), aged at 40 °C for 24 h, cooled to room temperature and placed in a reserved dryer. The core sand of the experimental oil was weighed with an 8 g electronic balance and pressed into thin sheets using a four-column press. The active surface agent of 100 mL, with different concentrations of surfactant (0.1%, 0.2%, 0.3%, 0.5%; mass fraction), the same concentration (0.3%, mass fraction) and different synthetic proportions (SDBS, SDBS-1, SDBS-2, SDBS-3, SDBS-4), was added to the oil-displacement meter, and the oil-displacement meter was placed at 50 °C. In the constant-temperature water bath, the data was recorded and the displacement efficiency was calculated until the displacement volume remained unchanged [30,31,32,33]. 

## 4. Conclusions

Four products were obtained (denoted as SDBS-1 to SDBS-4) via synthesis with sodium dodecylbenzene sulfonate as the main component. The structures of the surfactants were characterized with mass spectrometry and infrared spectroscopy. The results showed that the expected products were obtained. The CMC values changed after the reaction. The foaming abilities of SDBS to SDBS-4 were tested, the interfacial tension of oil and water was tested, and the emulsifying abilities were tested. The results showed that the foaming abilities of SDBS-1 to SDBS-4 are better than that of SDBS, the foam-decay mechanism was all liquid drainage and the interfacial tension of oil and water could be reduced. SDBS-1 has a strong emulsifying ability at low and high concentrations. The emulsion formed is also enhanced after reaction. Only the emulsion particles formed by SDBS-4 increased with increasing surfactant concentration. The oil-displacement efficiencies of SDBS-1 to SDBS-4 are obviously higher than that of SDBS, and the oil-displacement efficiency of SDBS-2 is the best, at 25%. The above experimental results all indicate that these products have an excellent ability to reduce oil–water interfacial tension, and they can be used in the oil and petrochemical industry for oil production and have certain practical uses.

## Figures and Tables

**Table 1:** Critical micelle concentrations and surface tensions, at 0.5%, of SDBS to SDBS-4.

Surfactant	CMC(%)	Surface Tension (0.5%) (mN/m)
SDBS-1	0.0600	31.392
SDBS-2	0.0353	33.471
SDBS-3	0.0529	32.359
SDBS-4	0.0637	31.274

**Table 2:** The “Φ” values of SDBS to SDBS-4 foams at different times.

Φ Value	0 min	10 min
SDBS	0.670	0.867
SDBS-1	0.673	0.871
SDBS-2	0.683	0.878
SDBS-3	0.671	0.870
SDBS-4	0.678	0.873

**Table 3:** Syneresis rates of emulsions produced by SDBS to SDBS-4 in different concentrations.

Surfactant	Water-Splitting Rate	
	0.1%	0.2%
SDBS	75	0
SDBS-1	0	0
SDBS-2	63	0
SDBS-3	58	0
SDBS-4	0	75

**Table 4:** Oil-displacement rate with the concentration of 0.3% wt at 50 °C.

Surfactant	SDBS	SDBS-1	SDBS-2	SDBS-3	SDBS-4
Oil-Displacement Rate %	10	15	25	15	20

**Table 5:** Names of synthesized products.

Reactant A	Reactant B	Raw-Material Ratio (n/n)	Solvent	Product Number
SDBS	Formaldehyde (40% solution)	1:0	Methanol	SDBS
		1:1		SDBS-1
		1:2		SDBS-2
		1:3		SDBS-3
		1:4		SDBS-4

## Data Availability

The data presented in this study are available wholly within this manuscript.
